# The effect of laser-assisted incubation on bovine *in vitro* embryos

**DOI:** 10.3389/fvets.2026.1828209

**Published:** 2026-08-04

**Authors:** Wei Zhang, Ying Chen, Jiaxin Zhang, Gulmira Abudureyimu, Hong Dong, Jiapeng Lin, Liqin Wang

**Affiliations:** 1 Xinjiang Key Laboratory of Reproductive Regulation and Breeding for Ruminant Livestock, Xinjiang Academy of Animal Sciences, Urumqi, China; 2 Institute of Biotechnology, Xinjiang Agricultural University, Urumqi, China; 3 College of Animal Science, Xinjiang Agricultural University, Urumqi, China

**Keywords:** blastocyst rate, cattle, hatchability, *in vitro* embryos, laser-assisted incubation

## Abstract

**Introduction:**

This paper aimed to enhance the hatching of bovine in vitro produced embryos by exploring their optimal LAH perforation/thinning pattern.

**Methods:**

Ovaries were col-lected from an abattoir to obtain cumulus–oocyte complexes. After in vitro fer-tilization, 4-cell, 8-cell, morula, and blastocyst-stage embryos were allocated to experimental groups, and their hatching outcomes were compared following four Laser-Assisted Hatching (LAH) treatments, which aim to facilitate embryo hatching from the zona pellucida and subsequent implantation into the endo-metrium by creating small pores or thinning the zona pellucida. The treatments included removal of ^1^/_4_ of the zona pellucida, removal of ^1^/_3_ of the zona pellucida, thinning of ^1^/_4_ of the zona pellucida, and thinning of ^1^/_3_ of the zona pellucida.

**Results:**

The results showed that: ① The hatching rate of 4-cell embryos with ^1^/_4_ zona removal was significantly higher than that of the control group (*p* < 0.01); ② the hatching rate of 8-cell embryos with removal of ^1^/_3_ of the zona pellucida was significantly higher than that of the control group (*p* < 0.01); ③ for morula-stage embryos, both ^1^/_3_zona removal and ^1^/_3_zona thinning resulted in significantly higher hatch-ing rates than the control (*p* < 0.01); and ④ the hatching rate of blastocysts with removal of 1/3 was significantly higher than that of the control group (*p* < 0.01).

**Conclusion:**

In conclusion, the most effective LAH treatments were ^1^/_4_zona removal for 4-cell embryos and ^1^/_3_zona removal for 8-cell, morula, and blastocyst-stage embryos, all of which yielded improved hatching rates.

## 1 Introduction

*In vitro* Embryo Production (IVP) technology is fundamental to modern animal husbandry, enabling the rapid propagation of superior breeds, the preservation of genetic resources, and biotechnological research (1). According to data from the International Embryo Transfer Society (IETS), China has a huge potential market for embryo technology. However, compared with *in vivo* embryos, those produced *in vitro* generally suffer from developmental arrest, quality decline, and low blastocyst hatching rates (2), which currently restrict the efficiency and economic benefits of IVP technology.

Blastocyst hatching is a critical developmental process before embryo implantation, as the embryo must successfully penetrate the zona pellucida (ZP) to contact the endometrium and initiate implantation (3). The zona pellucida is an elastic, non-cellular matrix approximately 13–15 μm thick that envelops the surface of an oocyte or embryo (4). It plays a crucial role in processes such as fertilization and embryo hatching as it is devoid of cellular material (5, 6), allowing the passage of many small molecules. However, transport efficiency decreases as the zona pellucida thickens. Studies have shown that *in vitro* culture can lead to physical or chemical alterations in the zona pellucida of bovine embryos, such as thickening, hardening, or reduced elasticity, significantly increasing the difficulty of and decreasing the likelihood of spontaneous embryo hatching. This has also become one of the key factors contributing to suboptimal pregnancy rates following *in vitro* embryo transfer (7–9).

Assisted Hatching (AH) technology emerged as a solution that involves artificially thinning or disrupting the zona pellucida of embryos to facilitate hatching (10). This can be achieved by mechanical, chemical, or laser treatment. Among these, Laser-Assisted Hatching (LAH) is currently the most widely used due to its short exposure time, precise targeting, simplicity of operation, and absence of physical or chemical contact (11, 12). It involves utilizing the precise photothermal effect of laser beams at specific wavelengths to efficiently and controllably create openings or thinning regions on the zona pellucida, typically at sites of greater zona pellucida space or higher fragmentation to reduce mechanical resistance during embryo incubation (13).

Although mechanical incision of the zona pellucida is simple, it may have profound effects on embryonic development, such as damaging the inner zona pellucida, microbial invasion and infection of the embryo, causing the cleavage sphere to lose its independence (14), altering the embryonic transcriptome and affecting metabolic activities (15), and increasing the miscarriage rate of suboptimal frozen–thawed embryos after processing (16). However, most studies indicate that this technology has positive effects, such as accelerating embryo hatching through early exposure to critical growth and metabolic factors (17–19) facilitating *in vivo* hatching with artificial openings (20); and aiding in the treatment of recurrent implantation failure (21, 22). Existing research primarily focuses on the effects of LAH on pregnancy and effects on human embryos after cryopreservation and thawing, while systematic studies and applications in the field of *in vitro* embryo production in livestock, such as cattle, remain relatively scarce.

Therefore, this study aims to provide a scientific basis for establishing an efficient, safe, and standardized laser-assisted incubation protocol for bovine embryos by comparing developmental outcomes at different stages under various treatment modalities. This research offers robust technical support for enhancing the potential and practical application value of bovine *in vitro* embryos.

## 2 Materials and methods

### 2.1 Experimental reagents and equipment

The egg extraction, IVM, IVF, sperm washing, and IVC solutions used for *in vitro* embryo production are all Bredin® Bovine *In Vitro* Embryo Culture series products (Xinjiang Academy of Animal Husbandry, Institute of Biotechnology, Urumqi, Xinjiang, China). The IVF and sperm washing solutions are the BO base solution and SOF base solution systems, respectively. All other biochemical reagents were produced by Sigma (China) unless otherwise indicated. Equipment included a carbon dioxide incubator (ESCO, Singapore), a stereomicroscope (Nikon, Japan), and a laser membrane rupture instrument (Nikon).

### 2.2 Experimental design

This study performed laser-assisted hatching (LAH) treatments using an ILS-400 M semiconductor laser system designed for zona pellucida perforation. The system was equipped with a 1,480 nm pulsed laser light source. A fixed wavelength was uniformly adopted for all zona pellucida manipulation procedures on embryos at every developmental stage throughout the experiment, with no adjustments made to the wavelength parameters, to maintain consistent experimental conditions across all groups. The specific laser-assisted incubation methods are shown in Table 1, categorized into Drilling Laser-Assisted Incubation (D-LAH) and Thinning Laser-Assisted Incubation (T-LAH).

**Table 1:** Laser-assisted hatching with different treatment methods.

Group	^1^/_3_ zona pellucida	¼ zona pellucida
D-LAH	LD	SD
T-LAH	LT	ST

According to this table, the groups are referred to as LD, Long drilling group; LT, Long thinning group; SD, Short drilling group; and ST, Short thinning group, respectively.

The system lacked integrated software for automated calibration of the zona pellucida circumference and automatic segmentation into ^1^/_3_ or ¼ arcs. Therefore, a standardized manual workflow was established to control the ablation region. Manipulations were carried out under constant microscopic magnification. An ocular micrometer was used to estimate the full circular perimeter of the zona pellucida. Virtual evenly spaced reference points were applied to artificially divide the zona circumference into three or four equal arcs. Laser pulse ablation was restricted strictly to one designated arc segment to achieve removal of approximately ^1^/_3_ or ¼ of the zona pellucida. All laser positioning and ablation procedures were performed by a single experienced technician to minimize visual estimation bias. Treated blastocysts were subsequently cultured *in vitro* for further analysis.

**Figure 1:**
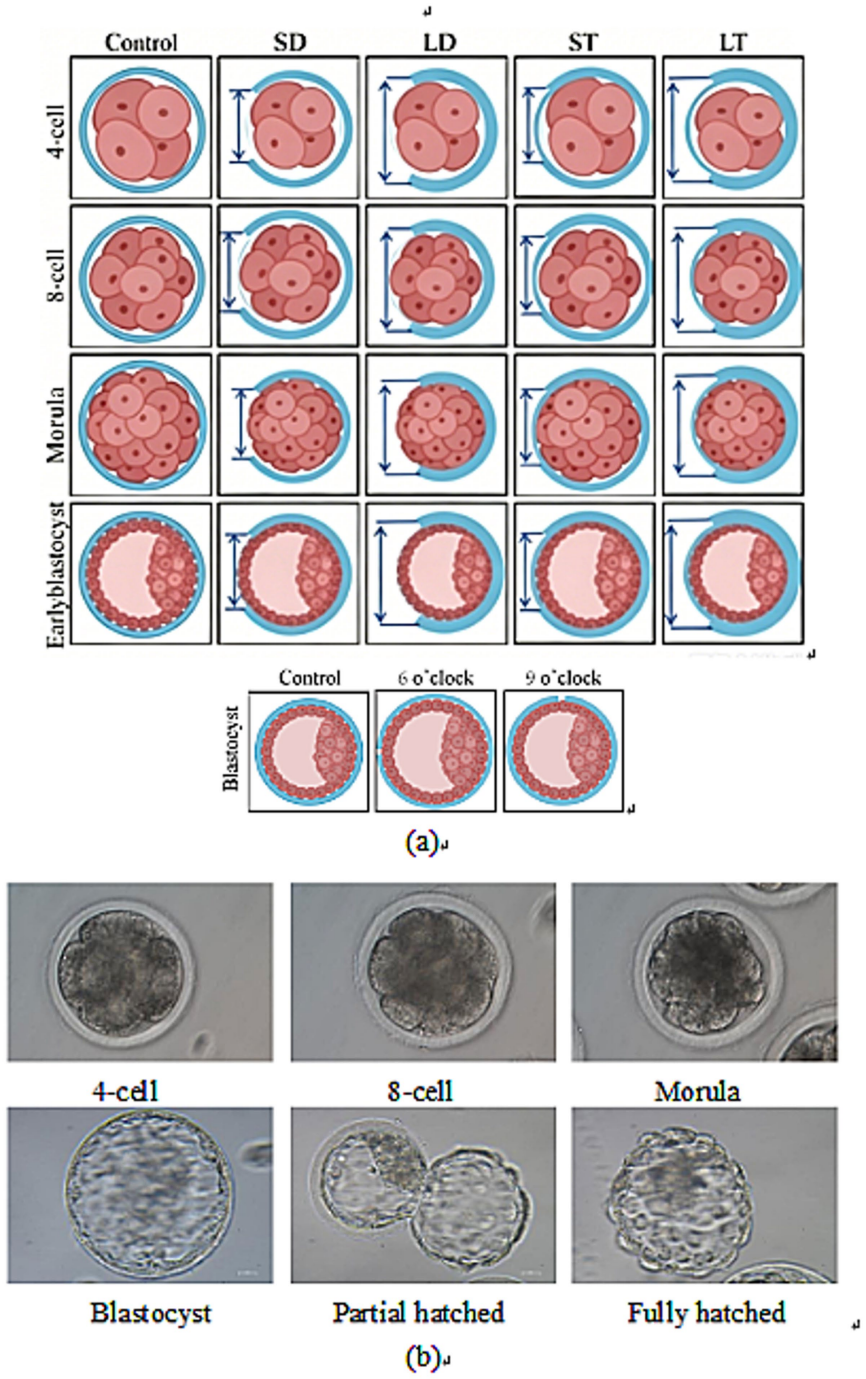
Different operations of laser-assisted incubation (40×, Scale: 50 μm). Effects of four AH treatments on the developmental stages of *in vitro* bovine embryos. **(a)** Schematic representation of AH treatments at different embryonic developmental stages, including four-cell, eight-cell, morula, early blastocyst and expanded embryos. **(b)** Representative pictures of four-cell, eight-cell, and morula embryos, as well as blastocyst and hatched embryos.

The D-LAH procedure penetrates the zona pellucida, while the T-LAH procedure covers ^1^/_3_ to ^1^/_2_ of the zona pellucida, with the laser range spanning ^1^/_3_ to ¼ of the zona pellucida circumference. According to Table 1, the groups are referred to as LD group, LT group, SD group, and ST group, respectively. These four LAH procedures were applied to four-cell, eight-cell, morulae embryo, and early blastocyst stages, with untreated embryos serving as the control group (Figure 1a). Representative images of four-cell, eight-cell, morulae embryo, expanded embryo, partially hatched, and fully hatched embryos are shown in Figure 1b.

### 2.3 Embryo collection and culture

#### 2.3.1 *In vitro* maturation of oocytes

Freshly slaughtered bovine ovaries with normal morphology were harvested from Shengyuan slaughterhouse in Changji City, Xinjiang, and placed in a thermos flask containing saline with added penicillin and streptomycin at 25 ~ 30 °C, and sent to the laboratory within 2 h. In the lab, the ovaries were gently washed with preheated saline (containing penicillin and streptomycin) at 30 °C until free of blood and water, and the follicular contents of 2–8 mm on the surface of the ovary were aspirated with a sterile 10 mL disposable syringe and a 0.9-gauge needle. The aspirated contents were collected in a 60-mm Petri dish, and oocyte complexes (COCs) with more than five layers of oocyte envelope and a uniform cytoplasmic colour without vacuoles were picked up under the stereoscope.

The collected oocytes were washed three times with the extraction solution, and then washed three times with the Bredin® Bovine IVM culture solution developed and produced in the laboratory. The four-well plate was pre-equilibrated for more than 4 h by adding 600 μL of IVM solution to each well, where after the washed COCs were transferred to the well-equilibrated IVM solution at 50 COCs/well. Plates were placed in the incubator for maturation culture at 38.6 °C, 5.0% CO₂ with saturated humidity for 22–24 h.

#### 2.3.2 *In vitro* fertilisation of oocytes

Two centrifuge tubes with 5 mL/tube of sperm washing solution, an appropriate amount of fertilising solution, and 440 μL/well four-well plates were equilibrated in a carbon dioxide incubator for 4 h before preparation of the oocytes.

After the COCs were matured and cultured *in vitro* for 22-24 h, a 100 μL pipette was set to pipette 60 μL, and the COCs with expanded oocytes were transferred into a large droplet of the pre-equilibrated IVF liquid. The same transfer was performed three times to adequately remove the IVM liquid around the oocytes. The extended oocytes were finally transferred to four well plates with 440 μL/well IVF fluid, also pre-equilibrated for more than 4 h, at 50 oocytes/well, and placed into an incubator to allow for fertilisation.

Frozen sperm were thawed in a 39 °C water bath, added to the preheated sperm washing solution in a pointed-bottom centrifuge tube, and mixed well. The sperm samples were centrifuged at 1500 r/min, for 4 min and the supernatant was removed before adding 5 mL of preheated sperm washing solution and mixing. The samples were centrifuged a second time and supernatant removed to leave about 300 μL of sample, which was gently flicked to mix before adding to the wells of sperm fertilisation solution for 18 h at the required concentration (sperm-to-egg ratio: 10,000-20,000:1). The sperm-egg mixtures were incubated for 18 h to allow for fertilisation.

#### 2.3.3 *In vitro* culture of fertilised eggs

After co-incubation, eggs were presumed fertilized and were washed three times with IVC solution pre-equilibrated for more than 4 h. After washing, the oral pipette was used to gently blow away the surrounding dead sperm and partially encapsulated oocytes, and the remaining fertilized oocytes were transferred into the IVC culture wells (600 μL/well) according to their experimental grouping and incubated at 38.6 °C, 5.0%CO_2_, 5.0% O₂ and saturated humidity. At 48 h after fertilisation, oocytes without cleavage were removed and the remaining samples air-sealed, incubated, and cultured until the seventh day after fertilisation to calculate the rate of blastocyst development ((number of blastocysts/number of cleavage)*100%).

### 2.4 Immunofluorescence (IF) assay

Embryos were fixed with 4% formaldehyde for 15 min, followed by permeabilization with 0.3% Triton X-100 for 10 min. Samples were then blocked with 5% goat serum at room temperature for 30 min. Subsequently, embryos were incubated with HSP70 rabbit polyclonal antibody (Catalog No. A12948SP, Abclonal, 1:100 dilution) overnight at 4 °C. This antibody was originally validated for human, mouse and rat samples. Considering the high evolutionary conservation of HSP70 among mammals, this antibody was applied to bovine *in vitro* embryos in the present study.

After three washes with PBST, the samples were incubated with ABflo® 647-conjugated goat anti-rabbit IgG (H + L) secondary antibody (Catalog No. AS060, Abclonal, 1:500 dilution) for 1 h at room temperature in the dark, and then rinsed three times with TBS buffer. Afterwards, the embryos were stained with DAPI (4′,6-diamidino-2-phenylindole, 1:10,000) for 30 min at room temperature away from light. After thorough washing three times with TBS buffer, the samples were mounted with mounting medium. Fluorescence images were observed and captured using a Nikon fluorescence microscope (Japan).

### 2.5 Terminal deoxynucleotidyl transferase biotin-dUTP nick end labeling (TUNEL) assay

TUNEL staining was performed using the TUNEL cell apoptosis detection kit (Biosharp, Beijing, China). In brief, samples were fixed with 4% formaldehyde for 20 min and permeabilized with 1% Triton X-100 at room temperature for 5 min. Subsequently, samples were incubated with streptavidin-TRITC working solution at 37 °C for 30 min under light protection. For positive controls, samples were pre-treated with DNase I reaction solution at 37 °C for 30 min, followed by labeling with TdT enzyme reaction solution for 1 h in the dark. Negative samples were labeled directly after permeabilization. Finally, samples were counterstained with DAPI e (1:1000) and fluorescence images were captured using a fluorescence microscope (Nikon, Japan).

### 2.6 Statistical analysis

Each experiment was replicated three times. Significant differences between blastocyst rates were detected by one-way ANOVA with Tukey’s multiple *post hoc* comparison test in GraphPad Prism 10.1.2 (GraphPad Software Inc., San Diego, CA, United States). Results are expressed as mean ± SD. *p* > 0.05 indicates no significant difference; *p* < 0.05 indicates a significant difference; *p* < 0.01 indicates an extremely significant difference.

## 3 Results

### 3.1 Embryonic development and hatchability of 4-cell embryos

The results are shown in Table 2, after four AH treatments of 4-cell embryos, the blastocyst rate was lower than that of the control group, except for the thinning ^1^/_3_ group. The remaining three treatment groups showed higher blastocyst rates than the control group, although only the thinning ¼ group was highly significant (*p* < 0.01). However, the hatching rate was significantly increased in all four treatment groups (*p* < 0.01), where the removal ¼ group had the highest hatching rate (up to 70.28%).

**Table 2:** *In vitro* developmental results of 4-cell bovine embryos after different AH treatments.

Groups	Embryo count	Blastocyst count	Blastocyst rate^a^	Partial hatchings count	Partial hatchability^b^	Complete hatching count	Full hatchability^c^	Total incubation rate^d^
Control	64	32	49.72 ± 2.65^BCb^	7	21.77 ± 0.92^Bb^	4	12.16 ± 1.64^Bb^	33.93 ± 2.46^C^
SD	71	37	52.28 ± 1.46^ABab^	14	37.91 ± 1.89^Aa^	12	32.36 ± 3.68^Aa^	70.28 ± 1.84^A^
LD	61	31	50.98 ± 0.98^ABb^	11	35.00 ± 3.47^Aab^	9	29.44 ± 2.42^A^	64.45 ± 2.22^ABa^
ST	61	36	59.05 ± 0.50^Aa^	8	22.14 ± 1.49^Bb^	7	19.37 ± 1.41^ABab^	41.51 ± 0.83^Bc^
LT	61	25	40.95 ± 0.50^Cc^	7	27.86 ± 1.49^ABb^	6	24.52 ± 2.49^ABa^	52.38 ± 2.38^Bb^

^a^(blastocyst count/embryo count) *100%; ^b^(number of partially incubated embryos/blastocyst count) *100%; ^c^(number of fully hatched embryos/blastocyst count) *100%; ^d^[(partial incubation count + complete incubation count)/blastocyst count]*100%. *n* represents the number of experimental replicates (*n* = 3), with each replicate containing ≥20 embryos. Different capital letters in the same column indicate statistically significant differences (*p* < 0.01); different lowercase letters indicate significant differences (*p* < 0.05).

### 3.2 Embryonic development and hatchability of 8-cell embryos

The results are shown in Table 3, after the 8-cell groups were treated with the four AH treatments, the blastocyst rate of the removal ^1^/_3_ was extremely significantly higher than that of the control group (*p* < 0.01), while the blastocyst rate of the remaining three treatment groups was not statistically different from that of the control group (*p >* 0.05). All four treatment groups showed extremely significantly higher hatching rates than that of the control group (*p* < 0.01), among which the removal ^1^/_3_ group was extremely significantly higher than that of the remaining three treatment groups (*p* < 0.01).

**Table 3:** *In vitro* developmental results of 8-cell bovine embryos after different AH treatments.

Groups	Embryos count	Blastocyst count	Blastocyst rate^a^	Partial hatchings count	Partial hatchability^b^	Complete hatchings count	Full hatchability^c^	Total incubation rate^d^
Control	60	31	51.67 ± 1.67^ABb^	6	19.39 ± 0.61^b^	6	19.39 ± 0.61	38.79 ± 1.21^C^
SD	60	28	46.67 ± 4.41^B^	9	32.24 ± 6.14^ab^	7	25.29 ± 7.68	57.54 ± 2.50^B^
LD	60	47	78.33 ± 4.41^Aa^	21	45.27 ± 6.03^a^	14	29.03 ± 7.40	74.30 ± 1.50^A^
ST	60	27	45.00 ± 7.64^B^	7	27.58 ± 6.03^ab^	8	28.97 ± 2.41	56.55 ± 3.62^B^
LT	60	27	45.00 ± 2.89^B^	7	26.57 ± 5.50^ab^	9	32.78 ± 4.34	59.35 ± 2.03^B^

^a^(blastocyst count/embryo count) *100%; ^b^(number of partially incubated embryos/blastocyst count) *100%; ^c^(number of fully hatched embryos/blastocyst count) *100%; ^d^[(partial incubation count + complete incubation count)/blastocyst count]*100%. *n* represents the number of experimental replicates (*n* = 3), with each replicate containing ≥20 embryos. Different capital letters in the same column indicate statistically significant differences (*p* < 0.01); different lowercase letters indicate significant differences (*p* < 0.05).

### 3.3 Embryonic development and hatchability of morulae embryos

The results are shown in Table 4, all four AH treatments at the morulae embryo stage showed statistical differences from the control in blastocyst and hatching rates. The blastocyst rate of all four treatment groups was highly significantly higher than that of the control group (*p* < 0.01), with the removal ^1^/_3_ group being highly significantly higher than that of the other three treatment groups as well (*p* < 0.01). The hatching rate of all four treatment groups was highly significantly higher than that of the control group (*p* < 0.01), with the removal ^1^/_3_ group and the thinning ^1^/_3_ group being highly significantly higher than that of the other two treatment groups as well (*p* < 0.01).

**Table 4:** *In vitro* developmental results of bovine morulae embryos after different AH treatments.

Groups	Embryos count	Blastocyst count	Blastocyst rate^a^	Partial hatchings count	Partial hatchability^b^	Complete hatchings count	Full hatchability^c^	Total incubation rate^d^
Control	61	30	49.21 ± 2.99^C^	7	23.47 ± 3.47	4	13.1 ± 2.56^Bb^	36.56 ± 1.93^C^
SD	61	39	63.97 ± 1.03^B^	13	33.33 ± 2.56	13	33.33 ± 2.56^Ba^	66.67 ± 2.56^B^
LD	64	53	82.78 ± 1.47^A^	11	20.76 ± 1.43	33	62.4 ± 1.36^A^	83.16 ± 2.56^A^
ST	62	39	62.88 ± 1.49^B^	13	33.27 ± 1.43	12	30.89 ± 1.38^Ba^	64.17 ± 1.48^B^
LT	63	42	66.62 ± 0.92^B^	9	22.03 ± 8.22	25	59.04 ± 6.48^A^	81.06 ± 1.83^A^

^a^(blastocyst count/embryo count) *100%; ^b^(number of partially incubated embryos/blastocyst count) *100%; ^c^(number of fully hatched embryos/blastocyst count) *100%; ^d^[(partial incubation count + complete incubation count)/blastocyst count]*100%. *n* represents the number of experimental replicates (*n* = 3), with each replicate containing ≥20 embryos. Different capital letters in the same column indicate statistically significant differences (*p* < 0.01); different lowercase letters indicate significant differences (*p* < 0.05).

### 3.4 Embryo development and hatchability of early blastocysts after different assisted hatching treatments

The results are shown in Table 5, when treating embryos at the early blastocyst stage, all four AH treatments showed statistical differences in hatching rates. The hatching rates of all four treatment groups were highly significantly higher (*p* < 0.01) than those of the control group, with the removal of ^1^/_3_ group being highly significantly higher (*p* < 0.01) than the other three treatment groups.

**Table 5:** *In vitro* developmental results of early bovine blastocysts after different AH treatments.

Groups	Embryos count	Partial hatchings count	Partial hatchability^b^	Complete hatchings count	Full hatchability^c^	Total incubation rate^d^
Control	62	13	21.03 ± 1.98^B^	9	14.45 ± 2.61^Bc^	35.48 ± 1.40^C^
SD	61	17	27.86 ± 1.48^B^	14	22.94 ± 1.51^ABbc^	50.79 ± 0.79^B^
LD	62	24	38.73 ± 0.63^A^	22	35.48 ± 1.40^Aa^	74.21 ± 1.43^A^
ST	61	14	22.94 ± 1.51^B^	16	26.19 ± 3.12^ABab^	49.13 ± 2.18^B^
LT	62	16	25.79 ± 1.43^B^	15	24.21 ± 0.40^ABb^	50.00 ± 1.37^B^

^a^(blastocyst count/embryo count) *100%; ^b^(number of partially incubated embryos/blastocyst count) *100%; ^c^(number of fully hatched embryos/blastocyst count) *100%; ^d^[(partial incubation count + complete incubation count)/blastocyst count]*100%. *n* represents the number of experimental replicates (*n* = 3), with each replicate containing ≥20 embryos. Different capital letters in the same column indicate statistically significant differences (*p* < 0.01); different lowercase letters indicate significant differences (*p* < 0.05).

### 3.5 Embryo development and hatchability of expanded blastocysts

The results are shown in Table 6, results found that both experimental groups showed a highly significant increase in the hatching rate as compared to the control group (*p* < 0.01). For the complete hatching rate, the 3/9 o’clock position point removal group showed highly significantly higher rates than the control and 6 o’clock groups (*p* < 0.01), while the 6 o’clock group had a significantly higher rate than the control group (*p* < 0.05).

**Table 6:** *In vitro* developmental results of bovine dilated blastocysts after different AH treatments.

Groups	Embryos count	Number of embryos hatched	Full hatchability^c^	Complete hatchings count	Total Incubation Rate^d^
Control	61	21	28.97 ± 2.41^Bb^	6	34.37 ± 2.36^B^
Drilling at 3/9 o’clock positions	60	43	67.46 ± 2.10^A^	29	71.67 ± 1.67^A^
Drilling at 6 o’clock positions	61	47	40.56 ± 3.06^Ba^	19	77.06 ± 1.51^A^

^a^(blastocyst count/embryo count) *100%; ^b^(number of partially incubated embryos/blastocyst count) *100%; ^c^(number of fully hatched embryos/blastocyst count) *100%; ^d^[(partial incubation count + complete incubation count)/blastocyst count]*100%. *n* represents the number of experimental replicates (*n* = 3), with each replicate containing ≥20 embryos. Different capital letters in the same column indicate statistically significant differences (*p* < 0.01); different lowercase letters indicate significant differences (*p* < 0.05).

### 3.6 Thermal effects of assisted incubation on embryos

**Figure 2:**
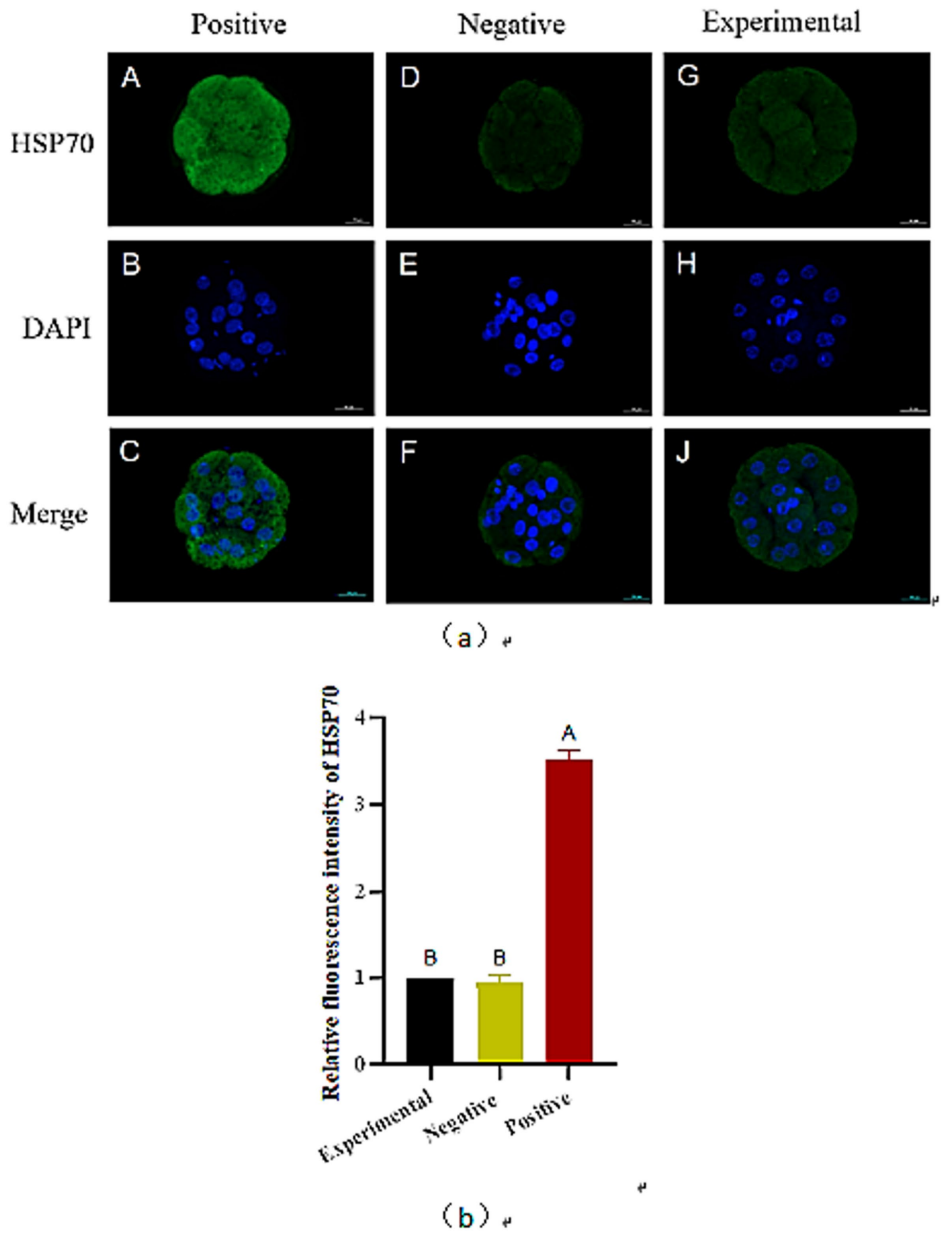
Effect of laser-assisted incubation treatment on heat stress of bovine *in vitro* embryos. **(a)** HSP70 **(A–J)** and DAPI **(B–H)** staining and merge **(C–J)** in three groups of *in vitro* bovine embryos. Typical embryos of each group are presented in the figure. Magnification is 40×, scale: 50 μm. **(b)** Relative fluorescence intensity of HSP70 in three groups of embryos. Capital letters indicate highly significant differences (*p* < 0.01), while lowercase letters indicate significant differences (*p* < 0.05). The same applies to the figure below.

The HSP70 fluorescence staining of the 16-cell stage embryos undergoing LD treatment was analyzed in positive controls, negative controls, and experimental embryos. The results are shown in Figure 2, all three groups showed positive HSP70 expression, indicating basal synthesis of this protein in the embryos. However, fluorescence intensity varied across groups, where the negative control (Figure 2F) and experimental groups (Figure 2J) exhibited similar fluorescence intensity, while the positive control group (Figure 2C) demonstrated significantly increased HSP70 protein fluorescence, suggesting elevated protein levels. Notably, no enhanced fluorescence was observed in individual cells.

### 3.7 Apoptosis

**Figure 3:**
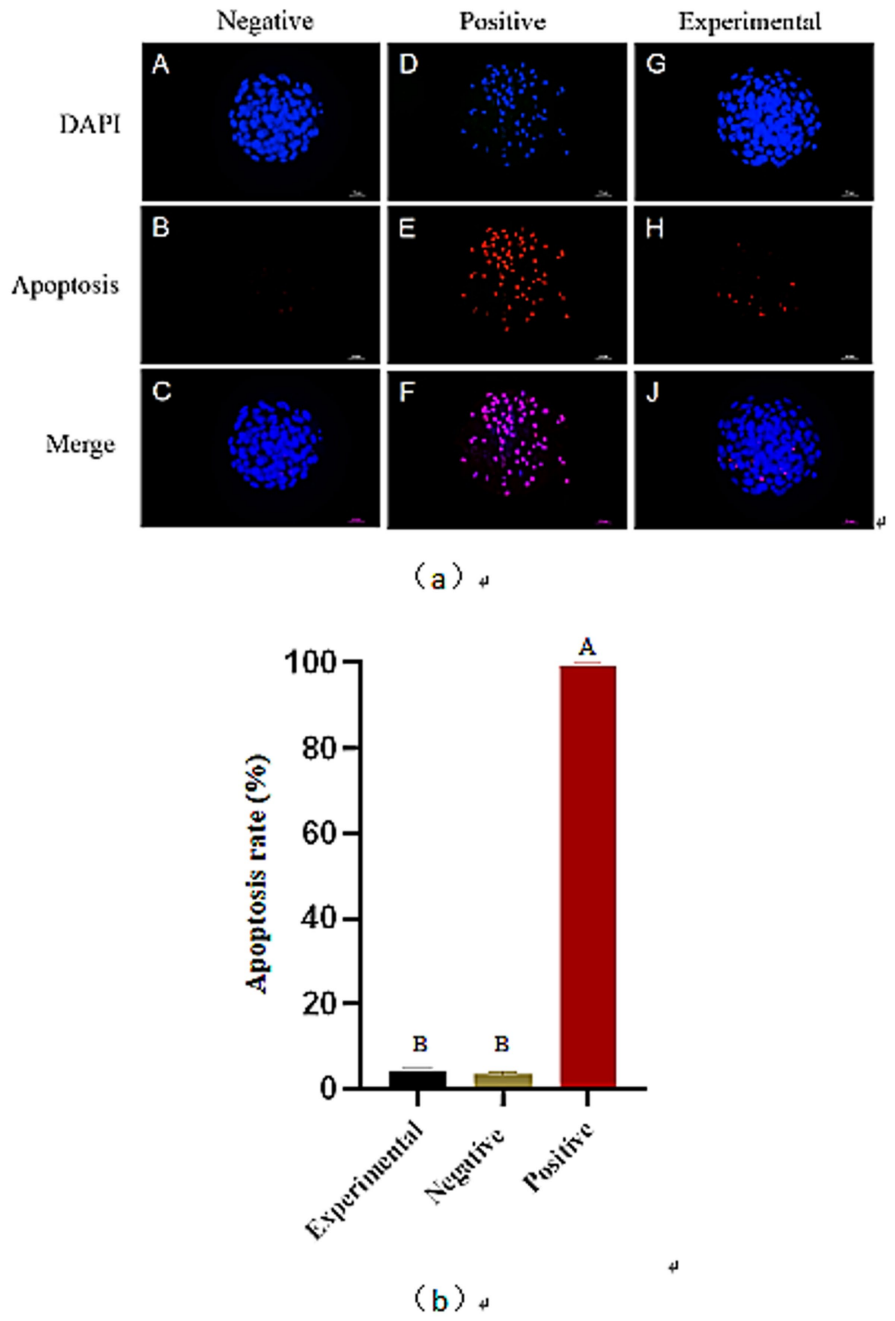
Effect of laser-assisted incubation treatment on apoptosis of bovine in vitro embryonic cells. **(a)** Apoptosis **(B,E,H)** and DAPI **(A,D,G)** staining and merge **(C,F,J)** in three groups of *in vitro* bovine embryos. Typical embryos of each group are presented in the figure. Magnification is 40×, scale: 50 μm. **(b)** Apoptosis rate of embryonic cells in the three groups.

TUNEL apoptosis detection was performed on morulae embryo samples, with the experimental groups treated with laser membrane disruption (LD). Comparative analysis of apoptosis rates was performed across positive controls, negative controls, and experimental embryos, and DAPI staining was employed for nuclear visualization. The results are shown in Figure 3, the results showed that the apoptosis rate of embryos in the experimental group (Figure 3J) was significantly different from the positive control group (Figure 3F), but similar to the negative control group (Figure 3C) This indicates that although laser zona drilling does not produce a significant pro-apoptotic effect.

## 4 Discussion

Currently, the pregnancy rate of *in vitro* produced bovine embryos is suboptimal after transfer, mainly due to the low blastocyst hatching rate. Laser-assisted hatching is widely applied in human assisted reproductive technology (ART) due to its convenience and efficacy in treating human infertility, which has led to the exploration of its potential combination with bovine intracytoplasmic sperm injection (IVP) technology to improve *in vitro* hatching rates. Previous studies have demonstrated that the hatching rate of LAH groups was significantly higher than that of the control group, suggesting high efficiency. Filatov et al. (23) proved that femtosecond laser-assisted LAH surgery was effective and did not induce heat shock protein production in embryos, and Liu et al. (24) demonstrated that the developmental outcomes of preschool-aged children born after LAH treatment were comparable to those of non-LAH groups, confirming its safety. D S et al. (25) used laser-assisted hatching to thaw frozen mouse embryos, increasing the hatching rate from 38.5 to 52.5%, and Baatarsuren et al. (26) showed that blastocyst transfer without the zona pellucida was more effective than complete embryo transfer. Maria et al. (19) found that LAH could promote early hatching of blastocysts without compromising quality. Weihai et al. (27) experimentally demonstrated that repeated LAH on day six increased the blastocyst formation rate on day seven and suggested that LAH could achieve higher proportions of total and viable blastocysts in low-grade cleavage-stage embryos (28), and Shuyuan et al. (3) reported that LAH improved the rate of high-quality blastocyst formation and hatching rates in non-advanced-age patients. In this study, laser-assisted hatching with laser-regulated (LD) treatment at different pre-hatching embryonic stages improved hatching results, with significantly improved hatching rates of LA-treated morulae embryos likely due to the size of the zona pellucida gap, which neither compromises embryo integrity nor hinders blastocyst extraction.

Notably, selecting the optimal embryonic stage for hatching remains a challenge. Zygotic gene activation occurs during the two-cell stage embryo (29), and manipulation of LAH at this stage may affect gene expression and subsequent embryonic development. Similarly, cells in the four-cell stage exhibit distinct developmental fates and efficiencies, particularly in the differential distribution of the Sox21 gene (30), where minor laser-induced damage can amplify adverse consequences in later developmental stages, and large-scale epigenetic modifications at this stage are even more susceptible to mechanical manipulation. In this study, the eight-cell stage demonstrated significantly higher blastocyst rates in the LD group compared to the control group, while other experimental groups showed slightly lower rates. This is primarily attributed to the limited number of early embryonic cells (4 to 8 cells), which are more vulnerable to laser thermal damage or shock waves disrupting cell integrity or interfering with critical developmental events (e.g., genomic activation). Additionally, the laser aperture can expose the embryo prematurely, exposing it to environmental stressors (e.g., osmotic pressure, toxins) or mechanical damage.

Although the early blastocyst treated with LAH showed inferior results compared to the morulae embryo, all hatching rates in the experimental group were significantly higher than those in the control group. Notably, the LD group exhibited a significantly higher hatching rate than the other three experimental groups, probably due to the blastocyst’s fluid-filled cavity and specialized cell layers (TE, trophoblast cells; ICM, inner cell mass) tightly adhering to the zona pellucida. Consequently, high-energy lasers are likely to directly damage the cell layers or collapse the blastocyst cavity. Therefore, the size, shape, and position of the blastocyst opening are critical. Given the unique characteristics of blastocysts in developing TE and ICM, particularly dilated blastocysts, the opening position and size were optimized. Specifically, point elimination was performed at two locations: close to the ICM (3/6 o’clock direction) and farther from the ICM (9 o’clock direction). The results showed that both experimental groups exhibited a highly significant increase in hatching rates compared to the control group. Regarding the complete hatching rate, the 3/9 o’clock position point removal group showed a significantly higher rates than the control and 6 o’clock groups, while the 6 o’clock group also showed a significantly higher rate than the control group.

Importantly, current results showed that morulae embryos exhibit more pronounced effects after LAH treatment compared to early embryos and blastocysts, with both blastocyst formation rates and hatching rates improving significantly in the LAH group. This may be attributed to the fact that morulae embryos have established extensive cell connections during the embryonic stage, forming a more compact structure that creates larger perivitelline spaces. Unlike blastocysts, these embryos do not adhere excessively to the zona pellucida, and any minor cellular damage can be compensated for by other cell clusters.

The effects of LAH treatment on subsequent embryonic development were validated through HSP70 (heat shock protein 70) and apoptosis staining. Heat shock proteins are conserved proteins activated in response to physiological and environmental stresses (such as heat shock and oxidative stress), and are crucial in cellular stress response mechanisms (31). Understanding changes in HSPs during stress is beneficial for animal husbandry, as animals rapidly synthesize HSPs as a self-protective mechanism. Apoptosis refers to the gene-controlled, autonomous, and orderly death of cells to maintain internal homeostasis, which is an active process aimed at better adaptation to the environment. Liu et al. (15) found that laser-assisted zona pellucida manipulation did not induce apoptosis or stress in mouse blastocysts. It should be noted that gene transcription and protein translation are regulated at distinct hierarchical levels; processes including post-translational modification and protein degradation can lead to inconsistencies between mRNA levels and protein abundance, and only protein levels were analyzed in the present study. Similarly, the staining results in this study showed no statistically significant difference in HSP70 protein expression levels between the experimental group and the negative control group. Laser perforation also did not cause apoptosis in blastocyst cells, validating the safety of this technique.

The present study confirmed that zona pellucida drilling directly exposes early embryos to external environmental stress. In addition, compromised structural integrity of the zona pellucida may exert potential adverse effects on the subsequent biosafety and cryopreservation of embryos: during the temperature ramping procedures of embryo cryopreservation and thawing, the protective function of the zona pellucida is weakened, which renders cell membranes susceptible to damage and reduces embryonic tolerance to osmotic fluctuations, thereby impairing the cryo-survival efficiency and subsequent developmental potential of embryos. Future research may further optimize operating protocols targeting this issue to mitigate associated risks.

The effects of laser-assisted hatching (LAH) on embryo implantation and offspring growth were not validated here through embryo transfer, and it is recommended for future studies. Fortunately, previous research has confirmed its feasibility in human embryos, supporting its potential use in bovines. Kexin (32) and colleagues, for example, discovered no noteworthy statistical variance in clinical pregnancy rates when comparing LAH to traditional laser-assisted hatching (LAH). Similarly, Chong et al. (33) indicated that LAH does not impact clinical outcomes, nor does it elevate the chances of miscarriage or ectopic pregnancy. Chaofeng et al. (34) demonstrated that LAH treatment increases the probability of live birth, while Ling et al. (35) suggested that prolonged culture of highly fragmented embryos with LAH could yield promising clinical results. Furthermore, Namath et al. (36) reported no negative impact of LAH on the implantation and pregnancy rates of fresh embryo transfers using vitrified donor oocytes, while Kanoi et al. (37) confirmed that LAH improves pregnancy outcomes in women undergoing vitrified embryo transfer, and Du et al. (38) suggested that LAH provides a potential pathway to enhance implantation. In general, laser-assisted hatching has demonstrated safety and promising results, potentially benefiting various patient groups, thus providing both theoretical backing and practical proof for bettering *in vitro* embryo implantation and successful pregnancies in cattle.

Abnormal mutations or dysregulated expression of growth-related imprinted genes IGF2 and IGF2R are key causes of large offspring syndrome induced by in vitro embryo technologies. Meanwhile, genes associated with lipid and glucose metabolism also directly regulate embryonic development and metabolic homeostasis of individuals. Restricted by experimental conditions and research duration, genetic analysis of the above genes was not performed in the present study. In future research, we will focus on IGF2, IGF2R and genes related to glucose and lipid metabolism. Combined with gene sequencing, expression quantification and other techniques, systematic genetic detection will be carried out to further evaluate the biosafety of the proposed technique and supplement the relevant research conclusions.

## 5 Conclusion

Current results recommend elimination of ¼ of the zona pellucida circumference for four-cell stage embryos, and ^1^/_3_ for eight-cell stage embryos, morulae, and early blastocysts. For expanded blastocysts, the 3/9 o’clock position point elimination method should be selected. Overall, morulae embryos treated with LAH demonstrated the best hatching rate improvement, increased blastocyst formation efficiency, with no heat shock response or apoptosis that could adversely affect subsequent development. This protocol significantly enhances the hatching rate of bovine embryos *in vitro* with excellent safety, providing critical technical support for the optimization and promotion of bovine IVP technology. Further research is warranted to identify optimal parameters and to validate their practical application in cattle and sheep.

## Data Availability

The original contributions presented in the study are included in the article/supplementary material, further inquiries can be directed to the corresponding authors.

## Glossary

LAH: Laser-Assisted Hatching
IVP: *In vitro* Embryo Production
ZP: zona pellucida
AH: Assisted Hatching
IVM: *In vitro* maturation
IVF: *In vitro* fertilization
IVC: *In vitro* culture
SD: Short region Drilling
LD: Long region Drilling
ST: Short region Thinning
LT: Long region Thinning
COCs: Cumulus-oocyte complexes
DAPI: 4′,6-diamido-2-benzidinol
TUNEL: TdT-mediated dUTP Nick-End Labeling
TRITC: Tetrame-thylrhodamine
TdT: Terminal Deoxynucleotidyl Transferase
ANOVA: Analysis of variance;
HSP: heat shock protein
ART: assisted reproductive technology
TE: trophoblast cells
ICM: inner cell mass
